# Understanding patients’ satisfaction with physician assistant/associate encounters through communication experiences: a qualitative study in acute hospitals in England

**DOI:** 10.1186/s12913-019-4410-9

**Published:** 2019-08-28

**Authors:** Francesca Taylor, Mary Halter, Vari M Drennan

**Affiliations:** Centre for Health & Social Care Research, Joint Faculty of Kingston University & St George’s University of London, St George’s University of London, Cranmer Terrace, London, SW17 0RE UK

**Keywords:** Physician assistants, Physician associates, Hospital patient encounters, Patient satisfaction, Clinician-patient communication, Qualitative research

## Abstract

**Background:**

Physician assistants/associates (PAs) are a recent innovation in acute hospital teams in England and many other countries worldwide. Although existing evidence indicates generally high levels of patient satisfaction with their PA hospital encounters, little is known about the factors associated with this outcome. There is a lack of evidence on the process of PA-patient communication in hospital encounters and how this might influence satisfaction. This study therefore aimed to understand patients’ satisfaction with PA acute hospital encounters through PA-patient communication experiences.

**Methods:**

A qualitative study was conducted among patients and representatives of patients seen by or receiving care from one of the PAs working in acute hospital services in England. Semi-structured interviews were undertaken face-to-face with study participants in the hospital setting and shortly after their PA encounter. Data were coded and analysed using thematic analysis. The study was framed within a theoretical model of core functions of medical encounter communication.

**Results:**

Fifteen patients and patient representatives who had experienced a PA encounter participated in interviews, across five hospitals in England. Four interrelated communication experiences were important to participants who were satisfied with the encounter in general: feeling trust and confidence in the relationship, sharing relevant and meaningful information, experiencing emotional care and support, and sharing discussion on illness management and treatment. However, many participants misconceived PAs to be doctors, raising a potential risk of reduced trust in the PA relationship and negative implications for satisfaction with their PA encounter. Participants considered it beneficial that patients be informed about the PA role to prevent confusion.

**Conclusions:**

PA encounters offer a constructive example of successful clinician-patient communication experiences in acute hospital encounters from the patient’s perspective. Study participants were generally naïve to the PA role. Hospital services and organisations introducing these mid-level or advanced care practitioner roles should consider giving attention to informing patients about the roles.

**Electronic supplementary material:**

The online version of this article (10.1186/s12913-019-4410-9) contains supplementary material, which is available to authorized users.

## Background

Acute hospital teams in England, as in many other countries worldwide, have seen the introduction of a new type of mid-level or advanced care practitioner in response to doctor shortages, the physician associate (PA), known as physician assistant in other countries [1, 2]. PAs are trained at postgraduate level to work supervised by a doctor within medical/surgical teams. Although PAs are not doctors, they have the skills and knowledge to deliver holistic care and treatment, undertaking physical examinations, investigations, and diagnosis across a wide range of clinical specialities [3, 4]. These mid-level practitioner types of roles are advocated by the World Health Organization to address medical workforce shortages [5].

PAs have a fifty-year history in the United States (US) and have been recently introduced to many more countries, including Canada, Ghana, India, the Netherlands, Germany, New Zealand and Saudi Arabia [2, 6]. As in most of these countries, PAs are still novel in the English setting, where the NHS is the main healthcare provider, funded by taxation and free at the point of delivery [7]. A small number of English and Scottish hospital organisations first recruited US-trained PAs to short-term pilot schemes in the mid-2000s [8]. United Kingdom (UK)-trained PAs entered the NHS workforce from 2009 [9]. Numbers remained very low until recently; there has been an increase in PA graduates from University courses, with education being supported by public funds. The aim is to have 1000 graduated by 2020 [1, 10].

The limited number of research studies that included reports of patients’ experiences of PAs in hospital outpatient settings in the US [11, 12], and inpatient settings in Canada [13, 14], the US [15, 16], the Netherlands [17], and England [18, 19], found generally high levels of patient satisfaction with their PA encounters. There is some evidence that patients appreciate PA communication in hospital encounters [13, 16, 18, 19]. However, there is lack of understanding about the process of PA-patient communication [20] and how this might influence satisfaction, particularly in the context of the acute hospital setting. This setting presents significant challenges to effective communication within clinician-patient encounters: their often brief duration; the absence of a prior relationship; and the likelihood that the acutely ill patient will have interacted with many different medical/surgical team staff members [21, 22].

### Theoretical framework: communication functions of effective medical encounters

Clinician-patient communication as an interactional process within medical encounters has been shown to affect a range of health care quality and safety outcomes, including patient satisfaction, treatment compliance, information understanding, and psychosocial wellbeing [23–26]. In this study, we adopted a theoretical approach based on the processes through which communication in medical encounters influences outcomes, to examine PA-patient acute hospital encounters. The evidence-based model of de Haes and Bensing [27] links communication functions considered best practice within a medical encounter to defined communication outcomes. It distinguishes six core communication functions: fostering the relationship(s); gathering information; delivering information; decision making; enabling disease and treatment related behaviour; and responding to emotions. It also differentiates between communication outcomes that are “immediate” (within the encounter), “intermediate” (shortly after the encounter), and “long-term” (over a longer time perspective), with patient satisfaction considered both an intermediate and long-term outcome. The six function model of medical communication is considered a helpful instrument to explore communication in medical encounters from the patient perspective, and to identify patients’ preferences and problems [28]. We used the theoretical framework to guide the study data analysis and interpretation.

Most extant evidence on these best practice communication functions is focused on medical encounters between patients and doctors. The literature indicates that positive patient satisfaction outcomes are linked to complex interrelationships among the different functions. Doctor-patient relationship development can be assisted by the generation of trust and communication of care [28, 29]. A positive and therapeutic relationship and patient-centred discussion, appear to facilitate information exchange [28, 30]. Empathetic communication also seems to support more accurate information gathering by doctors, clarity of information provision, and enhancement of patient trust [31–33]. Information exchange has been shown to encourage patient engagement in decision making [34, 35], and doctor response to patients’ emotions alongside information sharing may facilitate discussion on treatment management [30, 36].

These identified interrelationships reflect both the multifaceted nature of the communication concepts, and the interactive nature of doctor-patient communication within medical encounters [37]. Although similar issues are likely to apply to PA-patient encounters, there is a dearth of such data. Our study seeks to address this evidence gap by understanding patients’ satisfaction with PA acute hospital encounters through PA-patient communication experiences.

## Methods

### Study design and setting

A qualitative design was employed in the interpretive tradition to understand better the context and processes involved in PA-patient hospital encounters and enable insights into patients’ attitudes and opinions about PA communication behaviours [38, 39]. Guidance for ensuring quality when undertaking qualitative research [40, 41] was used to assess reliability and validity in designing an appropriate methodological approach, and data analysis and interpretation. The study was undertaken between March and May 2018 in five English hospitals which had recruited experienced US PAs to work for two years as ambassadors for the role and for the soon-to-graduate UK-trained PAs, as part of a workforce development programme [42]. The hospital study sites included one district general hospital, three urban teaching hospitals, and one urban speciality hospital, across four regions of England, with inpatient bed numbers ranging from 500 to 2500. There were no predefined range of areas or specialities involved.

### Participants

Eligibility criteria specified consenting adult patients - and adult representatives of patients – aged 18 years or over, in acute hospital services, seen by/receiving care from a US PA who worked in one of the participating study sites and volunteered to identify potential patient participants. Patients were excluded if they were not clinically stable and well enough to take part or lacking capacity to give informed consent. Sampling was purposive, designed to provide diversity of medical/surgical specialty of the PA and patient socio-economic group. Patients who met the eligibility criteria were identified by the volunteer PAs (five female PAs), who also made the initial enquiry about participation. Attempts were made to minimise selection bias by the researcher being present all day in a hospital ward/unit when recruiting participants, maximising the number of opportunities for eligible patients to be initially identified by a PA and then approached by the researcher. Patients expressing interest were given a study information sheet outlining the study purpose and what participation would involve, and asked whether they were willing to be introduced to a researcher. Patients could choose an individual personal interview, or nominate a friend or family member present in the hospital as a representative to be interviewed on their behalf.

A total of 18 patients and patient representatives were introduced to a researcher and 15 (83%) consented to be interviewed. One patient and two patient representatives withdrew before consenting due to the patient’s ill health.

### Data collection

Semi-structured interviews were undertaken face-to-face with participants in hospital during episodes of care or their use of hospital services, and shortly after the PA encounter. The interview consisted of a series of open-ended questions with supplementary prompts to allow key areas of interest to be explored without being overly prescriptive about content and direction [43]. An initial topic guide was developed based on the study aim, and informed by evidence on clinician-patient communication behaviours associated with patients’ positive experiences and satisfaction with medical encounters [27–37]. It was discussed with patient and public representatives, with whom we developed the guide further. It included questions about the participant’s perspective of the PA encounter through PA-patient communication experiences, their feelings about the care received, and their perspective on being attended by a PA in the future. The topic guide, including questions and questions’ prompts, is given as an Additional file 1.

One paired interview and 13 individual participant interviews were conducted. A minimum of one and a maximum of six participants were interviewed in each participating hospital study site. To ensure maximum confidentiality, nine interviews were undertaken in a room separate from the main inpatient ward, while five interviews took place at the bedside with curtain partition. Three patient representative interviews (one paired interview and two individual interviews) were undertaken with their family member (the patient) present. Interviews lasted 15 to 33 min (median 25). Interviews were audio-recorded, professionally transcribed, and the transcripts checked against recordings.

### Data analysis

All interview data were coded and analysed using thematic analysis [43]. The analysis was informed by the study topic guide and the theoretical framework of the study [27]. Transcripts from the first five interviews were read and re-read by one researcher (FT). Data were broken down using line-by-line coding and the codes clustered manually to identify preliminary categories based on issues and themes. These were scrutinised and discussed with a second researcher (VMD) who had also read the transcripts. The researchers asked theory-based questions of the data which assisted identification of category properties, for example, what prompted these emotions? An initial framework of themes was developed, together with a code book, and used to structure verbatim responses onto a spreadsheet. This process resulted in the development of 62 initial codes. The codes and themes were refined and elaborated collectively with data collection from further interviews. NVIVO V.11 software (QRS International) supported coding. As sequential analysis progressed, significant data were compressed to adhere around key analytic themes. Where data did not fit into existing themes, new ones were developed or existing ones modified until all data were coded by theme. This reflexive process [44] was undertaken independently by one researcher, supplemented by collaborative discussion with a second researcher before confirmation of themes.

## Results

There were 15 participants distributed across the five hospital study sites (Table 1). Eleven participants were patients and four were patient representatives. Two participants were recruited in the emergency department, four participants in acute medical units, three in paediatric units, and six in outpatient clinics. The participants were also recruited across different specialities (Table 2). The 11 patients interviewed ranged in age from 38 to 82 years. Three participants represented two patients aged less than 16 years. Eight of the participants were female and seven participants were male. For five participants English was a second language. Most participants had experienced a single PA encounter; three participants had two encounters.

**Table 1:** Study participants by hospital study site

Hospital study site	Number of study participants
Hospital 1	6
Hospital 2	3
Hospital 3	2
Hospital 4	3
Hospital 5	1

**Table 2:** Study participants by speciality

Speciality	Number of study participants
Acute internal medicine	4
Cardiology	3
Emergency medicine	2
Gastroenterology	3
Paediatric acute care	3

The data analysis process resulted in identification of four themes related to PA-patient communication: (1) Feeling trust and confidence in the relationship, with three sub themes - Perceived expertise, Perceived approachability, and PA perceived to be a doctor; (2) Sharing relevant and meaningful information, with three sub themes -Motivated to engage, Personalised explanation of health status, and Wanting to know about the PA role; (3) Experiencing emotional care and support; and (4) Sharing discussion on illness management and treatment. These themes contained elements associated with all six functions of the model of medical encounter communication [27] that framed this study; Table 3 maps the themes against the model.

**Table 3:** Communication elements within PA-patient encounters associated with core functions of medical encounter communication

Core functions of medical encounter communication (de Haes & Bensing, 2009) [27]	Fostering the relationship(s)	Gathering information	Decision making	Responding to emotions
		Providing information	Enabling disease & treatment related behaviour	
*Study themes*	*Feeling trust and confidence in the relationship*	*Sharing relevant and meaningful information*	*Sharing discussion on illness management & treatment*	*Experiencing emotional care and support*
	**PA conveying expertise:** “It was her demeanour. It was the way she explained things … She was very calm.” (Participant 3) “A good, professional manner... the way she was presented.” (Participant 6) **PA giving direct answer to questions:** “She was efficient and she knew, she wasn’t sort of telling you bull. She didn’t have to think before she gave an answer.” (Participant 10) **Personable approach of PA:** “She was friendly. It didn’t feel too official, which frightens mums a bit.” (Participant 1) “So we spoke a bit ‘Oh where did you go on holiday?’ a bit personal-like, it was just nice.” (Participant 2) **PA conveying respect:** “Treating patients like people...You can be efficient and still be pleasant and approachable.” (Participant 11)	**PA encouraging information sharing:** “She was finding out more about what’s wrong with him and help us understand what’s wrong with him.” (Participant 9) “She was very nice so I was happy to talk to her. She was very easy to talk to.” (Participant 2) **Participants feeling informed:** “She shared everything with us...I mean the problem.” (Participant 7) “She said ‘I found those kind of things and I’m going to go and see the senior doctor to talk about what we’re going to do’.” (Participant 8) “I was informed a bit more as well of what was happening. Just trying to get me a bed and trying to get the reduction in the blood pressure reading.” (Participant 4) **Participants understanding information provided:** “The person that we saw today, she was really nice and friendly and she even made it easier for us to understand what’s wrong with him.” (Participant 9)	**Participants understanding possible options:** “The score says what the risk factor is for me to have a heart problem … she went through that process and explained what my score is and what could be the next actions.” (Participant 12) “She looked through all my medical records, my previous records, and she tried to suggest the future course of action.” (Participant 14) **Shared discussion of management/ treatment options:** “I think she pulled out what was the important way ahead. Because patients have the choice and I think she helped me make the right choice.” (Participant 3) “We’ve decided not to look too deeply into whether there’s anything... that would cause mum distress to have a procedure done. And we’d just maintain her bloods and iron.” (Participant 1)	**PA communication of emotional support:** “It makes you feel more relaxed … it’s nice to have someone more informal.” (Participant 6) **PA demonstrating care:** “The way she approached us was kind, loving, and the way she treated my son, she was like someone that cared.” (Participant 8) “She is so lovely, she is so caring, she told me which will help me … Because she talks so politely, she make me understand everything.” (Participant 13) **PA enabling question asking:** “If someone is smiling at you and is very open to you, you can communicate.” (Participant 4) **PA listening skills** “She showed an interest in her patient, she listened, she was a listener.” (Participant 3)

### Theme 1. Feeling trust and confidence in the relationship

#### Perceived expertise

Trust in the expertise of the PA was a dominant consideration and the main criterion by which participants evaluated the quality of their encounter. Most participants perceived the PA that had attended them to have expertise and it was this expertise that formed the fulcrum of their confidence both in the PA and also the healthcare decisions resulting from the encounter.

Expertise was credited by participants based on a variety of different factors: personal attributes such as manner, presentation, and communication style, as well as specific skills and competencies.

*“She was polite, professional, to the point. She asked all the pertinent questions relating to my symptoms...She was familiar with all the medical terms.” (Participant 14, male, outpatient clinic)*

The general demeanour of the PA conveyed a sense of expertise for some participants. PAs were variously described as “professional”, “confident” and “calm”; these positive mannerisms were attributed by participants to enhanced confidence in the PA’s skills and knowledge.

*“She knew what she was doing or she put the impression she knew what she was doing which amounts to the same.” (Participant 10, female, emergency department)*

Displays of technical skill were also reported to inspire confidence in the PA. There were mentions of PAs successfully taking bloods, and in one consultation using a tool to calculate cardiovascular risk. However, more negatively, one participant commented on the PA experiencing difficulties with cannula insertion.

Additional confirmation of the PA’s expertise was reported by several participants through a senior doctor’s subsequent agreement with a PA’s diagnosis and suggested management plan. This was said to reinforce confidence and satisfaction with the encounter. Some participants described the PA leaving the encounter to refer to a doctor or consultant and then returning with their approval. There were no reports of the PA’s medical assessment being contradicted.

*“She knew what she was talking about and what she said to me is when she spoke to the doctor he agreed so she was quite confident. So she’s confident, I am.” (Participant 3, female, outpatient clinic)*

The efficiency of PAs was also recognised and valued, contributing to trust in their expertise. There were positive comments from participants about the PA being “to the point”, asking pertinent questions, and not wasting time. Several participants talked enthusiastically about PAs being informed and making things happen.

*“It seemed I got dealt with. We’d been in that room a long time, me and my friend. Left to the point where you get frustrated...Once I’d seen her it seemed I was more prioritised.” (Participant 4, male, acute medical unit)*

#### Perceived approachability

A personable approach was identified by many participants as a significant positive feature of the PA’s communication behaviour. PAs were frequently described as being pleasant and friendly as well as showing personal interest. Some participants commented appreciatively on being treated as an individual. This had been achieved by the PA for example, conversing about something other than the medical condition – such as the patient’s recent holiday. Another example occurred for a mother with her son in a paediatric ward, who recounted how the PA had demonstrated care beyond what was expected:

*“The way she cared about my son, was totally different … My son is sick. He was vomiting for the last five days, he doesn’t have any energy to sit, and she was the one who helped him to sit.” (Participant 7, female, paediatric ward)*

Several participants said they liked the PA, descriptors included “a people person”, “the human touch”, “kind”, and “lovely”. One participant attributed her positive sentiments to the PA’s gender:

*“I was just wondering...maybe I liked her because she was a lady. So maybe men would prefer the men one. I’m wondering if it’s a gender issue with me.” (Participant 3, female, outpatient clinic)*

#### PA perceived to be a doctor

Recognition among participants that their medical encounter had been with a PA was limited, with negative implications for trust in the PA for some participants. Only a few participants recalled being introduced specifically by title to a PA. Others were unaware the practitioner encountered was a PA until informed by the researcher. There was a lack of understanding about what a PA was among all participants. This latter result emerged after the researcher needed to clarify that the encounter had been with a PA, and participants were then asked: “What does the term physician associate mean to you?” and “What do you understand the physician associate role to be?” The term “physician associate” seemed unfamiliar to most participants and appeared to have little immediate meaning:

*“I’ve never heard of it before so I suppose I’m not quite sure what it involves.” (Participant 6, male, acute medical unit).*

Participants were unable to make an association between the title and the role PAs would perform within the medical/surgical team. This opaqueness also contributed to general confusion or misinterpretation of the PA status.

*“Well it’s the first time I’ve heard it today and I didn’t really know it. If I had to guess, a trainee physician, that’s the first thing which comes to mind.” (Participant 10, female, emergency department).*

Furthermore, many participants mistakenly understood their PA encounter to have been with a doctor. This misperception of PAs appeared to have been based on different contextual and experiential influences: wearing of civilian attire, type of medical procedure given, the PA’s communication of expertise and, their professional manner and confidence. It is likely that for some participants these impressions were underpinned by the anticipation they would encounter a doctor:

*“I just thought I was going to see a doctor for consultation.” (Participant 2, female outpatient clinic).*

A few participants expressed critical comments in response to learning from the researcher they had been attended by a PA. These negative sentiments were mainly linked to confusion about whether or not the PA encountered was a doctor or had completed their training. They seemed especially salient for participants seeking more control over their healthcare, who considered a doctor the most suitable clinician to attend them. Some intimations of concern were expressed about having been attended by someone not fully trained:

*“[The PA]‘s not a physician really … just not a medical doctor. How do we know [the PA] knows about anything or anything?” (Participant 5, female, emergency unit)*

Nevertheless, all but one of the participants said they would recommend receiving medical care from a PA. One participant thought it more appropriate patients see a “proper physician.”

### Theme 2. Sharing relevant and meaningful information

#### Motivated to engage

PAs were perceived by many participants to be accessible and to encourage conversation. Some participants seemed to feel that the PA created an opportunity for shared dialogue around their condition and treatment. A male participant explained how the PA had welcomed discussion around his medical history which had encouraged him to talk about previous scans undergone at a different hospital. Another participant, a daughter accompanying her mother in an outpatient clinic, described feeling confident about sharing information:

*“I felt that I could tell her how we’ve been coping and everything. I was quite happy to talk to her. I felt quite comfortable talking to her.” (Participant 1, female, outpatient clinic).*

However, a male inpatient who reported having seen several different staff in quick succession, could not recall discussing anything with the PA during their encounter:

*“Not a lot to be honest … I don’t remember answering.” (Participant 15, male, acute medical unit).*

#### Personalised explanation of health status

Provision of clear and informative explanations of the patient’s health status was an especially salient demonstration of the PA’s communication skills for many participants. For some participants these explanations provided meaning and connection between their treatment and where they were on their care pathway.

*“Explained what was happening going forward, the tests that would be taken and that sort of thing. She made it quite clear what was going to happen.” (Participant 6, male, acute medical unit).*

Plaudits were expressed for the PA giving the facts – openly, directly and, knowledgeably, with the accessible and comprehensible way PAs communicated mentioned by several participants. Use of straightforward language, that did not “bamboozle”, was praised. A female participant whose first language was not English spoke in complimentary terms about how the PA had sensitively adapted their communication to be better understood:

*“I feel quite comfortable to her. I couldn’t speak proper English, she – nicely explaining, understanding everything, and quite friendly.” (Participant 9, female, paediatric unit).*

Some participants made spontaneous favourable comparisons with doctor encounters, viewing PA encounters as less hierarchical, more friendly, and with provision of a more meaningful and personalised picture of the patient’s condition. This difference in approach to communicating information was described by one participant, a male inpatient, in the following way:

*“More of the human touch, not quite as formal … Hospitals can be quite unpleasant places so it’s nice to see someone with a more friendly face. Whereas doctors give you the nitty gritty, the associate tend to be more down to earth.” (Participant 6, male, acute medical unit)*

#### Wanting to know about the PA role

Many participants expressed the view that provision of information about PAs and their role prior to a hospital encounter would be beneficial to prevent confusion and misunderstanding. These data emerged after participants had been informed by the researcher that their encounter had been with a PA and were asked the probing questions: “How do you feel about what you have just heard? Why do you feel that way?” A male outpatient advocated that information about the PA be provided in the following way:

*“The letter didn't explain that... maybe that needs to be explained there is a new role. I mean it's not to bother any people, it's just to give some more understanding.” (Participant 12, male, outpatient clinic)*

Only one participant expressed the view it was unnecessary to know more about the role of the clinician they had encountered. They believed all NHS staff to be professionally trained and felt their experience of the PA encounter confirmed this assumption.

*“There was no doubt in my mind that she isn't professionally trained, right. So as such as a patient, I don't particularly see any reason why I want to know any more about her role.” (Participant 14, male, outpatient clinic)*

### Theme 3. Experiencing emotional care and support

A frequently described aspect of the PA encounters was how participants felt that the PA had made them more at ease, modifying any feelings of fear or nervousness. To illustrate their appreciation of how the PA’s approach had relaxed them, a female participant spoke about how frightening it can be to attend a hospital consultation:

*“I'm sure it makes all the patients feel comfortable and I'm sure that's what you need when you come into a strange hospital for a consultation. I think some patients might be quite terrified at the thought of consultations, but there was nothing terrifying about [PA].” (Participant 3, female, outpatient clinic)*

Several participants seemed to have experienced a sense of empathy, mentioning that the PA conveyed interest and understanding of what they had to say. The listening skills of the PA were referenced with particular appreciation by one participant.

Feeling emotionally comfortable in their interactions with the PA appeared to have motivated several participants to open up and engage more in discussing personal feelings about their illness or condition. A few participants recounted feeling encouraged to disclose and discuss emotional as well as physical concerns:

*“She was just approachable. I could speak to her and go really personal, because it is kind of a personal thing that I’m here for...I could really talk to her.” (Participant 2, female, outpatient clinic)*

Being able to talk in this way was thought beneficial in that it offered the PA better insight into their health status, enabling provision of more appropriate care.

Some participants described how the encounter had made them feel emotionally cared for by the PA:

*“Because she talks so politely, she make me understand everything, what happened to me....She just tell me everything and that’s why I think she is very caring.” (Participant 13, female, outpatient clinic).*

However, a male participant who had earlier spoken about being attended by several different medical staff, reported that the PA other than being pleasant had made no identifiable contribution to their care.

*“She put a pleasant face on [things] which I suppose is useful but I don’t know what care she gave.” (Participant 15, male, acute medical unit)*

### Theme 4. Sharing discussion on illness management and treatment

Many participants described how their encounters with a PA had led to clarification or heightened understanding of the most appropriate way forward to treat or to manage their health condition. One likely contributory factor was that the perceived caring, patient-centred approach of the PA seemed to have facilitated participants to ask questions. Two participants said they were unlikely to have felt comfortable raising their questions with another health professional; one referencing consultants, and another nurses:

*“I felt I could ask questions which you don’t always feel comfortable with asking nursing staff.” (Participant, 4, male, acute medical unit)*

The opportunity was valued as it was described as having allowed better understanding of specific issues of concern around health status, prognosis, and treatment. Most participants who asked a PA questions reported that they were satisfied with the response.

For some participants, the process of shared discussion with the PA about their health status and prognosis seemed to have resulted in better understanding of the most appropriate disease management plan.

*“If you do this thing, it is better for me because I have diabetes in low age because I am now only 38 so I have diabetes type 2. So it’s a little bit risky for me.” (Participant 13, female, outpatient clinic)*

Another participant mentioned feeling better informed about the treatment rationale:

*“She explained things to me regarding iron that I didn't [previously] understand.” (Participant 1, female, outpatient clinic)*

Although all participants seemed accepting of any decisions made during their PA encounter, very few participants talked about sharing the decision making process. However, one participant illustrated how the PA had involved them in a medication-related decision after undertaking a cardiovascular risk assessment:

*“She went through the details explaining where I am. She was not pushing me to accept anything, was leaving me to decide … So she was engaging in that respect you know. Not taking action or pushing anything on me that you should do.” (Participant 12, male, outpatient clinic)*

Another participant mentioned being given a choice of treatment although this was described more as agreement with the PA’s recommendation than active participation in decision making.

## Discussion

Our study identified four themes that collectively illustrate how study participants, who were generally satisfied with their PA acute hospital encounter, experienced PA-patient communication behaviours. The themes were: feeling trust and confidence in the relationship; sharing relevant and meaningful information; experiencing emotional care and support; and sharing discussion on illness management and treatment. These communication experiences accorded with the functions identified by Haes & Bensing as underpinning successful medical encounter communication (Table 3). The study adds to existing evidence by providing better understanding of the process of PA-patient communication in acute hospital encounters and how this might influence the high levels of patient satisfaction with PA hospital encounters reported in the literature [11–19].

This study identified that the perceived expertise and efficiency communicated by PAs were critical attributes linked to feelings of trust and confidence in the clinician, which helped promote a therapeutic relationship. Positive interactions that were considered friendly and person-focused also contributed to relationship building. Principles of understanding and respect for the individual [45, 46] featured in participants’ accounts of what it felt like to be treated in a personalised way. Overall, the PA encounters were associated with some of the key attributes that the literature suggests patients want from patient-centred clinician interactions [47–49]. These attributes included emotional support and empathy, psychosocial and lifestyle exchanges, time taken to listen to the patient perspective, and tailored communication.

Our findings showed that PAs were considered by participants to have enabled them to feel informed and connected about their condition and treatment. Patient-centred information provision is recognised as complex because needs and preferences frequently vary dependent on personal characteristics and context [50]. Acute hospital encounters pose a particular challenge given the limited time generally available for processing information [51]. Yet despite most participants in this study experiencing a single hospital encounter, they reported PAs having successfully linked information provision about care management to participant need. Making the right estimations of information need has been associated with empathetic attitude, contributing to patient understanding of the information [32].

De Haes and Bensing identified decision making as one of the core functions of medical encounter communication [27] and the value of shared decision making, even in the emergency department, has been emphasised in the literature [35, 52]. Participants in this study described being involved in conversations about their treatment rather than actively reaching a shared decision. This may reflect the PA encounter being a single event for most participants. Shared decision making is also arguably more effective as a collaborative process over time than a one-off decision [52]. Additionally, patient preferences for involvement in decision-making can differ widely especially in the hospital setting [51, 53], which is likely to influence how clinicians interpret these preferences. In this study, most participants expressed appreciation with the PA for enabling a better understanding of their health condition and treatment, even without evidence of shared decision-making.

More negatively our findings revealed poor recognition and comprehension of the PA role. While PAs were seen as working within a medical/surgical team there was uncertainty around what the role was and how it fitted with other hospital professions. Participants were often confused by the title; its meaning was not immediately obvious and needed explanation. Many participants mistakenly understood their encounter to have been with a doctor not a PA. These findings are congruent with one other small study [19] indicating patient confusion about the PA role in the English hospital system. A study in the Netherlands, where the role is also unfamiliar, similarly reported that hospital staff thought patients were unaware whether they had seen a doctor or PA [54].

This study provides new evidence about the potential for some patients’ trust and confidence in PAs to be affected by lack of transparency, with possible negative implications for the PA-patient relationship and patient satisfaction with their PA encounter. The importance of building patient trust and confidence when introducing new roles and ways of working into a health service has been reported in the literature [55]. The training and qualifications of PAs were of interest to many participants in this study and mattered in particular to those who seemed more actively engaged in their healthcare [56]. Some participants expressed concerns when made aware of their misconception that PAs were doctors. These findings suggest a need for more explicit explanatory patient information about the role and competence of the profession. As PA roles are being introduced in many different countries, further investigation is required as to the most appropriate mechanisms for this information provision in different health care settings.

The study has some limitations. While we endeavoured to employ a purposive sample to provide maximum diversity, the use of PAs as gatekeepers may have resulted in selection bias and recruitment of a convenience sample. We reported mostly positive participant experience data regarding PA encounter satisfaction, although we also reported some concern and confusion about the PA title and role. Additionally, the participating hospital organisations and PAs were volunteers. Although we achieved a range of hospital sites, all the volunteer PAs were female, working in a restricted range of medical/surgical specialties that employ PAs. As a qualitative exploration the findings cannot be generalised, however, they do offer insights and theoretical framing for potential further testing in clinical settings. Future large-scale studies are needed to explore patients’ experiences and perspectives not only on communication, but also on PA tasks and roles in the acute hospital context.

## Conclusions

PA-patient encounters offer a constructive example of how to achieve satisfying medical encounters for patients within the acute hospital setting, based on four interrelated communication experiences: feeling trust and confidence in the relationship, sharing relevant and meaningful information, experiencing emotional care and support, and sharing discussion on illness management and treatment. Study participants were generally naïve to the PA role; many thought their encounter had been with a doctor. Given international policy support for the greater use of mid-level practitioners to address global health workforce shortages, it is likely that PAs and other mid-level practitioner roles will be introduced more widely. Hospital services and other organisations introducing these types of roles should consider systematic processes for informing patients about the roles; to limit possible confusion, and to maximise the range of health care quality and safety outcomes that are known to arise from successful clinician-patient encounters. Future research could help determine how patients want to be introduced to PAs in different countries and health care settings.

## Additional file

Additional file 1: Topic guide for semi-structured interviews*.* Topic guide for semi-structured interviews, including questions and questions’ prompts. (PDF 40 kb)

## Data Availability

De-identified datasets analysed in the current study are available from the corresponding author on reasonable request.

## Abbreviations

NHS: National Health Service
PA: Physician Assistant/Associate
UK: United Kingdom
US: United States
