# Supplementary material for: Understanding patients’ satisfaction with physician assistant/associate encounters through communication experiences: a qualitative study in acute hospitals in England
Source: BMC Health Serv Res. 2019 Aug 28;19:603. doi: 10.1186/s12913-019-4410-9 (PMC6712610; doi:10.1186/s12913-019-4410-9)
Supplement: Supplementary file 1 — Topic guide for semi-structured interviews. Topic guide for semi-structured interviews, including questions and questions’ prompts. (PDF 40 kb) [file 12913_2019_4410_MOESM1_ESM.pdf]

Topic guide for semi-structured interviews

- *Question*: Please tell me why you are here and the type of care you have received, without giving personal medical details?

  Question prompts: Which ward/departments have you spent time in? For how long?
- *Question*: When did you first meet the physician associate (name)?

  Question prompts: Did you meet the physician associate (name) on any other occasions?
- *Question*: Please describe what happened during your encounter/s with the physician associate?

  Question prompts: What did they do? What did they say? How did you respond? Did you discuss anything? Did you ask any questions? Were you given any advice/information? Were any decisions made?
- *Question*: How do you feel about the care you received from the physician associate during your encounter/s?

  Question prompts: Was there anything you particularly liked/disliked? Was there anything you thought particularly helpful/unhelpful? Was it what you expected or not? Was it the same or different from care received from other staff in the hospital?
- *Question*: What are your feelings about how the physician associate communicated with you?

  Question prompts: What was your general impression? Was there anything you particularly liked/disliked? Was there anything you thought particularly helpful/unhelpful? Overall, how satisfied were you?
- *Question*: If you were to need medical care in future, would you be content to be attended by a physician associate or would you prefer someone different?

  Question prompts: Please explain your reasons why?

- *Question*: Is there anything else you would like to say or to comment on?

Exploratory prompts for all questions: Why do you feel that way? Can you tell me a little more about that? Why is that? How do you feel about that? Is there anything else you can think of?
